# Identification and Validation of Reference Genes for Quantification of Target Gene Expression with Quantitative Real-time PCR for Tall Fescue under Four Abiotic Stresses

**DOI:** 10.1371/journal.pone.0119569

**Published:** 2015-03-18

**Authors:** Zhimin Yang, Yu Chen, Baoyun Hu, Zhiqun Tan, Bingru Huang

**Affiliations:** 1 College of Agro-grassland Science, Nanjing Agricultural University, Nanjing, Jiangsu, China; 2 Department of Plant Biology and Pathology, Rutgers University, New Brunswick, New Jersey, United States of America; Chinese Academy of Sciences, CHINA

## Abstract

Tall fescue (*Festuca arundinacea* Schreb.) is widely utilized as a major forage and turfgrass species in the temperate regions of the world and is a valuable plant material for studying molecular mechanisms of grass stress tolerance due to its superior drought and heat tolerance among cool-season species. Selection of suitable reference genes for quantification of target gene expression is important for the discovery of molecular mechanisms underlying improved growth traits and stress tolerance. The stability of nine potential reference genes (*ACT*, *TUB*, *EF1a*, *GAPDH*, *SAND*, *CACS*, *F-box*, *PEPKR1* and *TIP41*) was evaluated using four programs, GeNorm, NormFinder, BestKeeper, and RefFinder. The combinations of *SAND* and *TUB* or *TIP41* and *TUB* were most stably expressed in salt-treated roots or leaves. The combinations of *GAPDH* with *TIP41* or *TUB* were stable in roots and leaves under drought stress. *TIP41* and *PEPKR1* exhibited stable expression in cold-treated roots, and the combination of *F-box*, *TIP41* and *TUB* was also stable in cold-treated leaves. *CACS* and *TUB* were the two most stable reference genes in heat-stressed roots. *TIP41* combined with TUB and *ACT* was stably expressed in heat-stressed leaves. Finally, quantitative real-time polymerase chain reaction (qRT-PCR) assays of the target gene *FaWRKY1* using the identified most stable reference genes confirmed the reliability of selected reference genes. The selection of suitable reference genes in tall fescue will allow for more accurate identification of stress-tolerance genes and molecular mechanisms conferring stress tolerance in this stress-tolerant species.

## Introduction

Determination of gene expression patterns and quantitative levels is a commonly-used approach for discovering genes controlling plant traits in various plant species responding to different environmental factors [1–4]. Quantitative real-time polymerase chain reaction (qRT-PCR) is presently regarded as the most effective tool for quantifying gene expression levels and variations in gene expression related to plant growth and development and stress responses by combining high specificity and sensitivity with efficient signal detection [5,6]. However, the accuracy of qRT-PCR analysis is strongly influenced by the stability of reference genes, quantity and purity of the mRNA templates, enzymatic efficiency in cDNA synthesis, and PCR amplification [5,6]. Among those factors, the first consideration of qRT-PCR for data normalization should be the selection of reference genes as the internal control of stable expression under different experimental conditions [5]. The use of proper reference genes in qRT-PCR assay is critically important for the accuracy of qRT-PCR results.

Most of the traditionally-used reference genes in the past were cellular maintenance or housekeeping genes, such as actin (*ACT*), tubulin (*TUB*), elongation factor 1a (*EF1a*), glyceraldehyde-3-phosphate dehydrogenase (*GAPDH*) and 18s ribosomal RNA (*18S rRNA*) [1,7,8]. Recent studies have found that the transcription levels of house-keeping reference genes may vary with changing experimental conditions in different organs and tissues, and across different plant species [9,10]. Given the availability of microarray data, some new reference genes with highly-stable expression levels, such as SAND family protein (*SAND*), clathrin adapter complex subunit family protein (*CACS*), F-box/kelch-repeat protein (*F-box*), phosphoenolpyruvate carboxylase-related kinase 1 (*PEPKR1*) and TIP41-like family protein (*TIP41*) were identified in *Arabidopsis* [5], and the homologous genes of those new reference genes were found in other species using BLAST analysis based on transcriptome and EST sequences. Examples of reference genes identified as being stable in their expression level under different experimental conditions in some plant species include *CACS* for buckwheat (*Fagopyrum esculentum*) and cork oak (*Quercus suber*) [11,12] and *TIP41* for *Caragana intermedia* [9]. Some reference genes were identified as the stable internal control for qRT-PCR of certain tissues and experimental conditions, such as SAND for salt-treated roots in *Caragana intermedia* [9]. It is critically important to select stable references for different organs in specific plant species under various environmental conditions in order to accurately quantify expression levels of target genes using qRT-PCR.

To date, most studies of reference gene expression in plants have focused on model and crop species, but few studies have identified reference genes that are suitable for perennial grass species [10,13]. Tall fescue (*Festuca arundinacea* Schreb.) is the most widely-used cool-season species as forage and turf owing to its high quality and productivity, as well as a wide range of stress adaptation [14,15]. Tall fescue possesses superior drought and heat tolerance among cool-season perennial forage and turfgrass species and is typically used as model species to identify molecular mechanisms of stress tolerance in perennial grasses [3,14,15]. Identification of stable reference genes under different environmental conditions is imperative for efficient and effective molecular breeding and discovery of stress-related genes in tall fescue. Therefore, according to homologue comparison between tall fescue EST sequences and Arabidopsis microarray data, nine candidate reference genes, including the four traditional genes (*EF1a*, *ACT*, *GAPDH*, *TUB)* and five new genes selected from *Arabidopsis* (*SAND*, *CACS*, *F-box*, *PEPKR1*, and *TIP41*) were examined in this study. The objective of the study was to identify stable reference genes for qRT-PCR analysis of target-gene expression in leaves and roots of tall fescue exposed to salinity, drought, cold, and heat stress. The expression levels of a target gene, *FaWRKY1*, isolated from tall fescue were assessed to validate the effectiveness of the selected reference genes identified during the study.

## Materials and Methods

### Plant materials and treatments

Tall fescue (cv. Barlex) seeds were planted in pots filled with a mixture of sand and soil (1:1 by volume). Plants were maintained in a growth chamber (MT8070iE, Xubang, Henan) with 12-h photoperiod, 25/20°C (day/night)) and relative humidity of 60%. After 4 weeks of seeding, seedlings of uniform size were transferred into half-strength Hoagland’s nutrient solution and maintained in hydroponic culture for 7 d before imposing stress treatments. For salinity treatment, plants were grown in nutrient solution containing 200 mM NaCl. For drought treatment, plants were grown in nutrient solution containing 30% PEG6000 with-1.03 MPa osmotic potential. For cold treatment, plants were maintained in a cold chamber (Haier, Qing Dao, China) at 3°C. Heat stress was imposed in the growth chamber set at 45°C. Each treatment was replicated three times in three containers of nutrient solution containing NaCl (salinity) or PEG (drought) or three chambers for the cold or heat treatment. Leaves and roots of three plants per replicate were collected following stress treatment at 0, 1, 3, 6, 12, or 24 h, and immediately frozen in liquid nitrogen and stored at -80°C for further analysis.

### Total RNA Isolation and cDNA Synthesis

Total RNA was extracted from leaves and roots using the TaKaRa RNAiso reagent, and then treated with RNase-free DNaseI (TaKaRa), following the kit instructions. RNA concentration was measured with a spectrophotometer (NanoDrop 2000, Thermo, USA) at 230, 260 and 280 nm, and the 260/280 nm ratio within the range of 1.80–2.20 and 260/230 nm ratio approximately 2.00 were retained. The first-strand cDNA was synthesized based on 0.5 μg total RNA using M-MLV reverse transcription system (TaKaRa), according to the manufacturer’s instructions. The cDNAs were diluted 1:10 with nuclease-free water prior to the qRT-PCR analyses.

### Selection of Potential Reference Genes and Primer design

Arabidopsis nucleotide sequences from the potential reference genes served as a query sequence for a TBLASTX search of the tall fescue GenBank EST database. Nine candidate RGs (*EF1a*, *ACT*, *GAPDH*, *TUB*, *SAND*, *CACS*, *F-box*, *PEPKR1* and *TIP41*) were identified and corresponding EST accession numbers and gene ontologies were labeled in Table 1. Primer Premier 5.0 software was used for specific primers design of qRT-PCR, with melting temperature between 55–65°C, primer length between 19–24 bp, and amplicon lengths within 100–255bp (Table 1).

**Table 1:** Candidate reference genes and primer sequences.

Gene symbol	Gene name	EST GenBank Accession	Arabidopsis homolog locus	Primer sequences (forward/reverse)	Amplicon length (bp)
***ACT***	Actin7	GT038376	AT5G09810	AGATCAAGGTCGTTGCTCCA/CTCCCAGACTAGACGATACAGC	189
***EF-1a***	Elongation factor-1a	GT037588	AT5G60390	GCGTGACATGAGACAAACGG/AACAGCAGGAAAACTCCAGAC	198
***TUB***	Alpha Tubulin	GT051159	AT5G19780	ATGCTTTCGTCTTATGCCC/CTCTTGGTTTTGATGGTTGC	215
***F-box***	F-box/kelch-repeat protein	GT039249	AT5G15710	GCCAAATGTCTGGTGCTTAG/TCATCCGCTTCGTCTTCAA	101
***PEPKR1***	Phosphoenolpyruvate carboxylase-related Kinase 1	DT688788	AT1G12580	GAACATCCTCCTTGTCAGCA/CCTCATTGTAACCGCCAGA	155
***SAND***	SAND family protein	GT037941	AT2G28390	ACCCAAGATTTCGAGCTGTAT/AACCTAAACCTCACATATCTCCC	188
***TIP41***	TIP41-like family protein	DT690696	AT4G34270	GAACCAAGACACTATGCAAACA/GAAATACCACTATCCGCTAACTCA	162
***CACS***	clathrin adaptor complex subunit	GT044151	AT5G46630	TCGCTACATCACGAGGGCT/AACAGGATACGGGGGAAGAATA	255
***GAPDH***	glyceraldehyde 3-phosphate dehydrogenase	GT035008	AT1G13440	TGAGAAGGCAGCCACCTATG/TGCTGTCACCCTGGAAGTCA	125
***FaWRKY1***	WRKY DNA-binding protein30	GT037051	AT2G03340	ACCTTAGCAACAGTAACCAGAGG/GCCAGAATAAAAGCGAACCA	121

### qRT-PCR analysis

qRT-PCR was assayed with a LightCycler 480 II (Roche, Switzerland), using the LightCycler 480 SYBR I master reaction system (Roche, Switzerland). Each 15 μL reaction mixture contained 5 μL of diluted cDNA, 7.5 μL 2×SYBR I master mix, 0.4 μL each primer (10 μM) and 1.7 μL ddH_2_O. The reaction conditions included an initial denaturing step of 95°C for 10 min followed by 40 cycles of 95°C for 15 s, 58°C for 15 s, and 72°C for 30 s, after which a melt curve was produced at 60–95°C. Each qRT-PCR analysis was performed in triplicate.

### Stability analysis

qRT-PCR efficiencies for each treatment were evaluated with the software LinRegPCR [16,17]. Four programs, GeNorm [18], NormFinder [19], BestKeeper [20] and RefFinder (http://www.leonxie.com/referencegene.php) were used for the determination of stability for eight potential reference genes across all treatments following the developer’s instructions. For GeNorm and NormFinder, the quantification cycle (Cq) values were transformed into relative quantities using the formula 2^-ΔCq^, in which ΔCq = each corresponding Cq value—minimum Cq value. The expression stability measurement (M) was calculated by GeNorm based on the average variations of a particular gene against all the other control genes in their expression levels. The stability value calculated by NormFinder is to determine inter- and intra-group variation and lowest stability will be top-ranked. BestKeeper analysis based on untransformed Cq values was used to make comparisons of the coefficient of variance (CV) and the standard deviation (SD), and the lowest SD and CV were used as detection indexes for the most-stable reference genes. RefFinder used the data from GeNorm (M values), NormFinder (Stability values), BestKeeper (CV and SD) and ΔCq values.

### Validation of reference genes by expression analysis of *FaWRKY1* under abiotic stresses

The previous reports showed WRKY transcriptional factors were responsive to various biotic and abiotic stresses [21,22]. *FaWRKY1* (Accession number GT037051) encoding a WRKY transcription factor gene was screened from tall fescue EST library in GeneBank data [15]. For the validation of selected reference genes from qRT-PCR data, the expression level of *FaWRKY1* was analyzed using the most-stable reference genes and the most-varying reference genes under different treatments, which was calculated as 2^-ΔΔCq^ method. Three technical replicates were performed for each biological sample.

## Results

### Identification of PCR Amplicons, Primer Specificity, and Amplification Efficiency of qRT-PCR

**Fig 1:**
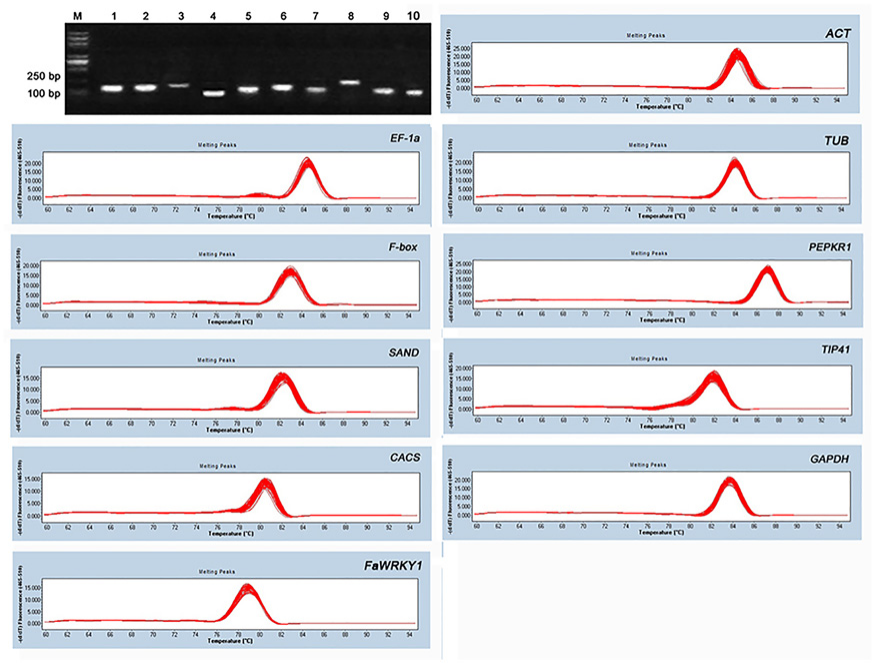
Primer specificity and amplicon size. Agarose gel (1.8%) electrophoresis indicates amplification of a single PCR product of the expected size for 10 genes (line 1–10: *ACT*, *EF1α*, *TUB*, *F-box*, *PEPKR1*, *SAND*, *TIP41*, *CACS*, *GAPDH* and *FaWRKY1*). Melting curves of 10 genes show single peaks. M represents 100 bp DNA marker.

The description of 10 genes, including nine candidate reference genes and one objective gene, primer sequences, and amplicon lengths are shown in Table 1. The primer specificities were confirmed by a single DNA band of RT-PCR products using agarose gel electrophoresis detection and single peak during melting curves assays of qRT-PCR (Fig. 1). The sequence of PCR amplicons were nearly identical (98–100% in similarity) to the corresponding EST sequences of tall fescue (data not shown). qRT-PCR efficiencies measured by LinRegPCR software for all 10 genes ranged from 1.91 to 1.96, representing acceptable efficiencies (1.8≤E≤2) [23] (Table 2).

**Table 2:** Amplification Efficiency of qRT-PCR of nine reference genes and a target gene.

Gene	SL*	SR	PL	PR	CL	CR	HL	HR
***ACT***	1.94±0.02	1.95±0.02	1.94±0.02	1.94±0.02	1.94±0.02	1.95±0.02	1.94±0.03	1.95±0.01
***CACS***	1.95±0.02	1.95±0.02	1.95±0.02	1.93±0.02	1.95±0.02	1.95±0.01	1.93±0.02	1.95±0.02
***EF1α***	1.94±0.02	1.91±0.02	1.95±0.02	1.92±0.02	1.91±0.04	1.93±0.02	1.94±0.02	1.94±0.03
***F-box***	1.94±0.02	1.94±0.03	1.94±0.03	1.93±0.02	1.93±0.02	1.94±0.02	1.94±0.02	1.94±0.02
***GAPDH***	1.92±0.02	1.96±0.02	1.94±0.02	1.95±0.02	1.93±0.02	1.94±0.03	1.93±0.02	1.96±0.02
***PEPKR1***	1.95±0.02	1.94±0.02	1.94±0.03	1.94±0.02	1.95±0.03	1.94±0.02	1.94±0.03	1.92±0.03
***SAND***	1.94±0.02	1.95±0.01	1.94±0.01	1.96±0.01	1.93±0.02	1.94±0.02	1.93±0.02	1.94±0.01
***TUB***	1.96±0.02	1.96±0.01	1.95±0.02	1.96±0.02	1.95±0.03	1.95±0.03	1.94±0.03	1.96±0.02
***TIP41***	1.95±0.02	1.93±0.02	1.93±0.02	1.92±0.02	1.92±0.02	1.90±0.03	1.94±0.03	1.92±0.02
***FaWRKY1***	\	1.92±0.02	1.92±0.03	\	\	\	\	\

*SR and SL: salt-treated roots and leaves, respectively; PR and PL: PEG-treated roots and leaves, respectively; CR and CL: cold-treated roots and leaves, respectively; HR and HL: heat-treated roots and leaves, respectively.

### Expression levels and variations of reference genes expressed as quantification cycle (Cq) values

**Fig 2:**
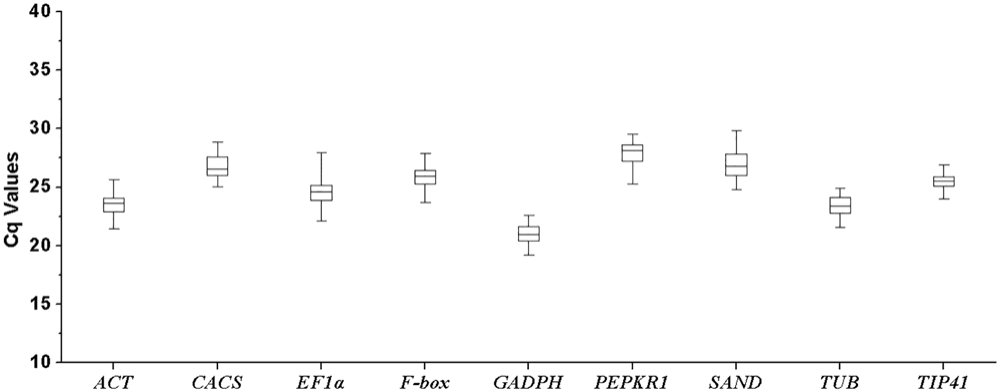
The quantification cycle (Cq) values of the candidate reference genes across all samples under four abiotic stresses. Lines across the Box-plot graph of Cq value represent the median values. Lower and upper boxes show the 25^th^ percentile to the 75^th^ percentile. Whiskers represent the maximum and minimum values.

The Cq values of nine reference genes were obtained by qRT-PCR analysis in 144 samples and variations of each gene were shown in the box-chart (Fig. 2). Cq values of all reference genes ranged from 19 to 30. Among nine candidate reference genes, *GAPDH* exhibited the highest expression level with a mean Cq of 21 and *PEPKR1* had the lowest expression level with a mean Cq of 27.9 (Fig. 2). The coefficients of variation (CV) (lower values represent lower variability) of nine reference genes were 2.55% (*TIP41*), 3.57% (*PEPKR1*), 3.58% (*F-box*), 3.75% (*ACT*), 3.82% (*GAPDH*), 3.88% (*CACS*), 3.94% (*TUB*), 4.45% (*SAND*) and 5.14% (*EF1α*).

### Stability of Candidate Reference Genes

#### 1. GeNorm analysis

GeNorm analysis was employed for stability assessment by the M values, which were defined as the mean variation of a gene compared to all others. The M values below the threshold 1.5 were considered to represent stable expression and lower M values indicate higher stability.

**Fig 3:**
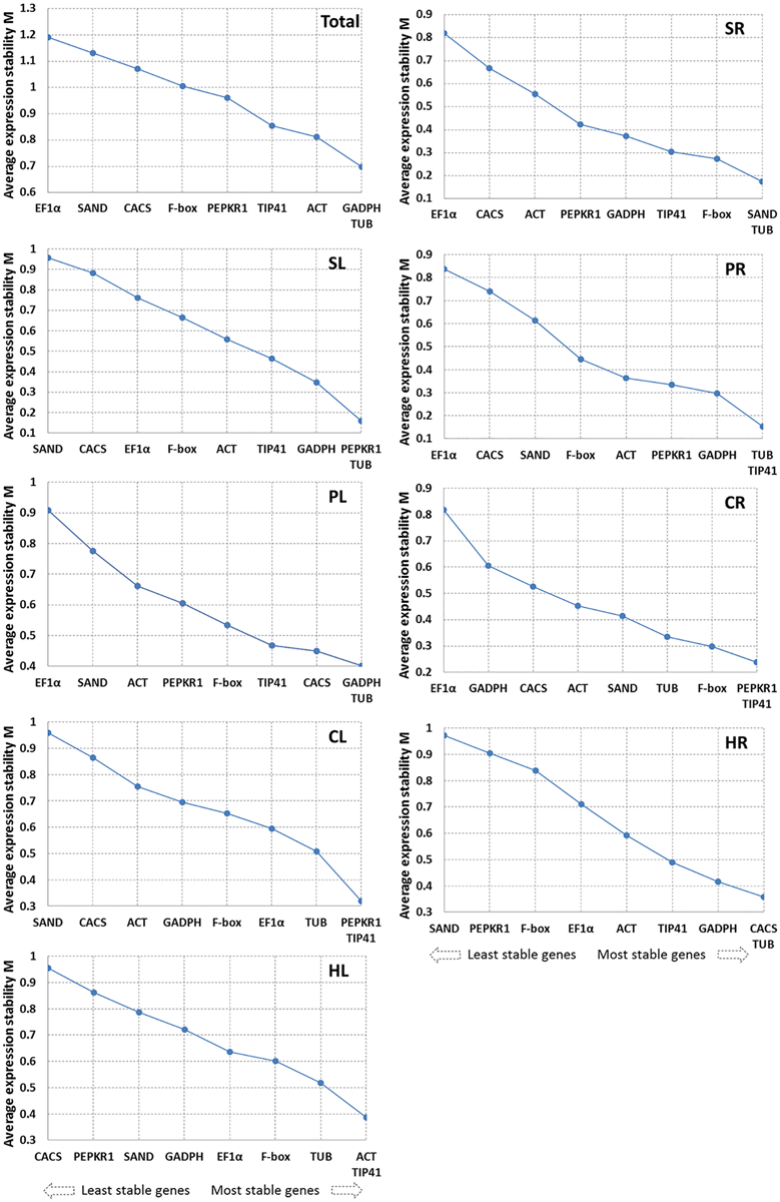
Gene expression stability values (M) and ranking of nine reference genes as assayed by GeNorm. The least stable genes are on the left and the most stable genes on the right. Total: all the treatments; SR and SL: salt-treated roots and leaves; PR and PL: PEG-treated roots and leaves; CR and CL: cold-treated roots and leaves; HR and HL: heat-treated roots and leaves.

The results showed that *GAPDH* and *TUB* with same M values were the two best reference genes for pooled samples including leaves and roots of all stress treatments or PEG-treated leaves (PL), *SAND* and *TUB* in salt-treated roots (SR), PEPKR1 and TUB in salt-treated leaves (SL), TUB and TIP41 in PEG-treated roots (PR), PEPKR1 and TIP41 in cold-treated roots and leaves (CR and CL), CACS and TUB in heat-treated roots (HR), and ACT and TIP41 in heat-treated leaves (HL). *EF1α* and F-box exhibited unstable expression in all samples (Fig. 3).

**Fig 4:**
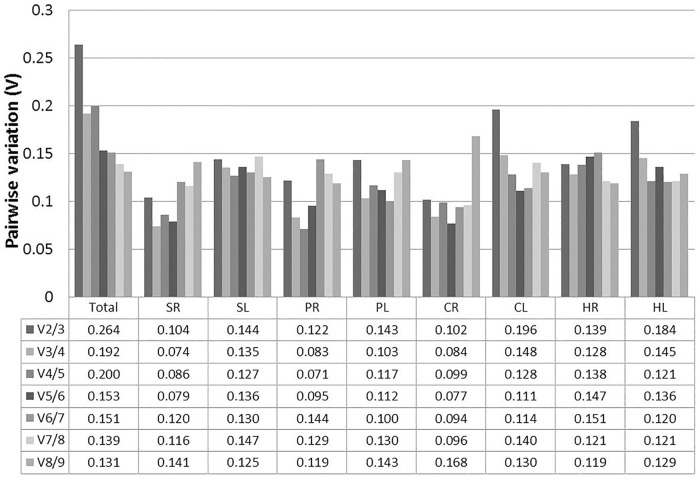
Pairwise variation (V) of the candidate reference genes calculated by geNorm. V_n_/V_n+1_ values were used for decision of the optimal number of reference genes.

In addition, the optimal number of reference genes required for accurate normalization was determined by the pairwise variation between ranked genes (V_n_/V_n+1_) following geNorm program. When a small variation appeared between V_n_/_n+1_ and V_n+1_/V_n+2_ or a V_n_ value was lower than the threshold of 0.15, the value (n) can be recommended as the optimal number of reference genes [18]. The V2/3 values for SR, SL, PR, PL, CR, and HR samples were lower than 0.15 (Fig. 4), indicating that two reference genes were suitable for normalization. Three reference genes were selected following the V3/4 values of CL and HL samples below 0.15. However, the value of 0.15 should not hold as a rigorous standard and higher cut-off values of V_n_/_n+1_ were found in several reports [12,24,25]. Our data shows slight variation between V3/4 (0.192) and V4/5 (0.200) in pooled samples, suggesting that three genes could be useful for normalization of all the samples.

#### 2. NormFinder analysis

The stability values of different reference genes were obtained through the NormFinder algorithm, in which lower values indicates higher stability (Table 3). The top three stable references genes were identified as *TIP41* (0.553), *ACT* (0.633) and *GAPDH* (0.634) for all samples using the NormFinder analysis (Table 3). *GAPDH*, *TIP41* and *TUB* were identified as the top three stable genes in PR samples while *TUB*, *TIP41* and *F-box* were ranked as top three in HL and CL samples. *TIP41* was the most-stable gene in SL and CR samples. *SAND* or *CACS* were separately ranked first for their stability in SR or HR samples respectively. *PEPKR1* was ranked second in CR samples and exhibited low stability in other samples. *EF1α* had the lowest rankings of stability in the pooled samples, SR, PR, PL and CR samples.

**Table 3:** Stability analysis of reference genes assayed by NormFinder software.

Rank	Total*	Stability	SR	Stability	SL	Stability	PR	Stability	PL	Stability	CR	Stability	CL	Stability	HR	Stability	HL	Stability
	Gene		Gene		Gene		Gene		Gene		Gene		Gene		Gene		Gene	
**1**	*TIP41*	*0*.*553*	*SAND*	*0*.*117*	*TIP41*	*0*.*246*	*GAPDH*	*0*.*153*	*GAPDH*	*0*.*2*	*TIP41*	*0*.*119*	*F-box*	*0*.*154*	*CACS*	*0*.*255*	*TUB*	*0*.*289*
**2**	*ACT*	*0*.*633*	*TUB*	*0*.*251*	*F-box*	*0*.*438*	*TIP41*	*0*.*217*	*F-box*	*0*.*281*	*PEPKR1*	*0*.*226*	*TUB*	*0*.*521*	*TUB*	*0*.*367*	*TIP41*	*0*.*315*
**3**	*GAPDH*	*0*.*634*	*F-box*	*0*.*342*	*TUB*	*0*.*49*	*TUB*	*0*.*344*	*TUB*	*0*.*361*	*F-box*	*0*.*28*	*TIP41*	*0*.*552*	*TIP41*	*0*.*435*	*F-box*	*0*.*418*
**4**	*TUB*	*0*.*788*	*TIP41*	*0*.*413*	*PEPKR1*	*0*.*626*	*ACT*	*0*.*367*	*TIP41*	*0*.*472*	*SAND*	*0*.*349*	*ACT*	*0*.*602*	*ACT*	*0*.*614*	*ACT*	*0*.*467*
**5**	*F-box*	*0*.*831*	*GAPDH*	*0*.*418*	*ACT*	*0*.*647*	*PEPKR1*	*0*.*426*	*CACS*	*0*.*513*	*TUB*	*0*.*359*	*GAPDH*	*0*.*648*	*GAPDH*	*0*.*707*	*EF1α*	*0*.*589*
**6**	*PEPKR1*	*0*.*931*	*PEPKR1*	*0*.*463*	*GAPDH*	*0*.*65*	*SAND*	*0*.*773*	*ACT*	*0*.*719*	*ACT*	*0*.*522*	*PEPKR1*	*0*.*675*	*EF1α*	*0*.*72*	*SAND*	*0*.*752*
**7**	*CACS*	*0*.*935*	*ACT*	*0*.*713*	*EF1α*	*0*.*986*	*F-box*	*0*.*802*	*PEPKR1*	*0*.*798*	*GAPDH*	*0*.*679*	*EF1α*	*0*.*709*	*F-box*	*0*.*93*	*GAPDH*	*0*.*937*
**8**	*SAND*	*1*.*075*	*CACS*	*0*.*915*	*CACS*	*1*.*007*	*CACS*	*0*.*989*	*SAND*	*0*.*94*	*CACS*	*0*.*736*	*CACS*	*0*.*988*	*PEPKR1*	*0*.*992*	*PEPKR1*	*0*.*941*
**9**	*EF1α*	*1*.*14*	*EF1α*	*1*.*257*	*SAND*	*1*.*099*	*EF1α*	*1*.*047*	*EF1α*	*1*.*27*	*EF1α*	*1*.*503*	*SAND*	*1*.*146*	*SAND*	*1*.*044*	*CACS*	*1*.*141*

*Total: pooled samples from all treatments;

SR and SL: salt-treated roots and leaves, respectively; PR and PL: PEG-treated roots and leaves, respectively; CR and CL: cold-treated roots and leaves, respectively; HR and HL: heat-treated roots and leaves, respectively.

#### 3. BestKeeper Analysis

BestKeeper program was used for the determination of standard deviation (SD) and the coefficient of variation (CV) of Cq values, with lower SD and CV representing higher stability. According to the ranking by BestKeeper (Table 4), the most stable genes were *GAPDH* for PL sample, *TIP41* for HR and HL samples, *PEPKR1* for SR and PR samples, and *F-box* was the most stable gene in SL, CR, and CL samples. *SAND* was ranked third for SR samples but exhibited the lowest rankings for PL, CL, and HR samples. *CACS* was ranked third in SL and PL samples and showed the lowest stability in HL samples. The stability of *ACT* was ranked second for stability in CL samples but ranked seventh in SR, SL, PL, and CR samples. *TUB* was ranked second in SR and HL samples, while *EF1α* was ranked low in stability in SR, SL, PR and CR samples.

**Table 4:** Stability analysis of reference genes assayed by BestKeeper software.

Rank	Total*	SR	SL	PR	PL	CR	CL	HR	HL
**1**	*TIP41*	*PEPKR1*	*F-box*	*PEPKR1*	*GAPDH*	*F-box*	*F-box*	*TIP41*	*TIP41*
**CV±SD**	1.98±0.50	1.80±0.51	0.89±0.23	0.76±0.21	1.38±0.29	1.68±0.44	1.44±0.37	1.00±0.25	1.17±0.30
**2**	*GAPDH*	*TUB*	*TIP41*	*F-box*	*TIP41*	*PEPKR1*	*ACT*	*CACS*	*TUB*
**CV±SD**	3.04±0.64	2.42±0.55	1.85±0.47	1.20±0.32	1.47±0.37	1.57±0.45	1.86±0.44	1.09±0.28	1.30±0.31
**3**	*F-box*	*SAND*	*CACS*	*ACT*	*CACS*	*TIP41*	*TIP41*	*ACT*	*F-box*
**CV±SD**	2.76±0.71	2.48±0.64	1.82±0.49	1.52±0.35	1.51±0.42	2.10±0.55	1.79±0.46	2.01±0.46	1.32±0.34
**4**	*ACT*	*F-box*	*TUB*	*GAPDH*	*F-box*	*TUB*	*TUB*	*TUB*	*ACT*
**CV±SD**	3.10±0.73	2.58±0.69	2.39±0.58	1.78±0.36	1.90±0.49	2.46±0.56	2.15±0.51	2.1±0.48	1.58±0.38
**5**	*TUB*	*TIP41*	*SAND*	*TIP41*	*TUB*	*GAPDH*	*GAPDH*	*GAPDH*	*EF1α*
**CV±SD**	3.37±0.79	3.16±0.81	2.49±0.66	1.50±0.38	2.15±0.52	3.05±0.63	2.64±0.56	2.43±0.50	2.18±0.53
**6**	*PEPKR1*	*CACS*	*PEPKR1*	*TUB*	*EF1α*	*SAND*	*EF1α*	*PEPKR1*	*SAND*
**CV±SD**	2.87±0.80	3.44±0.90	2.39±0.67	1.72±0.38	2.53±0.63	2.65±0.71	2.36±0.57	2.61±0.70	2.12±0.58
**7**	*CACS*	*ACT*	*ACT*	*SAND*	*ACT*	*ACT*	*PEPKR1*	*F-box*	*PEPKR1*
**CV±SD**	3.35±0.90	4.04±0.93	3.32±0.79	3.32±0.87	2.72±0.66	3.40±0.79	2.22±0.61	3.14±0.77	2.68±0.75
**8**	*EF1α*	*GAPDH*	*GAPDH*	*CACS*	*PEPKR1*	*CACS*	*CACS*	*EF1α*	*GAPDH*
**CV±SD**	3.83±0.95	4.54±0.94	4.02±0.85	4.11±1.08	2.81±0.77	3.02±0.81	2.84±0.76	4.03±0.95	3.62±0.77
**9**	*SAND*	*EF1α*	*EF1α*	*EF1α*	*SAND*	*EF1α*	*SAND*	*SAND*	*CACS*
**CV±SD**	3.65±0.98	4.50±1.09	4.11±1.02	4.54±1.13	3.50±0.99	5.69±1.44	3.36±0.93	4.91±1.30	3.28±0.89

*Total: pooled samples from all treatments;

SR and SL: salt-treated roots and leaves, respectively; PR and PL: PEG-treated roots and leaves, respectively; CR and CL: cold-treated roots and leaves, respectively; HR and HL: heat-treated roots and leaves, respectively. CV: Coefficient of Variation; SD, standard deviation.

#### 4. RefFinder analysis

RefFinder (http://www.leonxie.com/referencegene.php) was used to achieve the comprehensive rankings of candidate reference genes by integrating four common analysis programs (geNorm, Normfinder, BestKeeper and ΔCq method) [13,26]. According to the comprehensive analysis of RefFinder (Table 5), most-stable genes were *TIP41*, *GAPDH*, and *ACT* for all the samples, *SAND* and *TUB* for SR samples only, *TIP41* and *TUB* for SL samples only, *TIP41* and *PEPKR1* for CR samples, *F-box*, *TIP41* and *TUB* for CL samples, *GAPDH* and *TIP41* for PR samples, *GAPDH* and *TUB* for PL samples, and *CACS* and *TUB* for HR samples, and *TIP41*, *TUB* and *ACT* for HL samples.

**Table 5:** Most stable and least stable combination of reference genes based on RefFinder analysis.

Experimental treatments																	
Total		SR		SL		PR		PL		CR		CL		HR		HL	
Most	Least	Most	Least	Most	Least	Most	Least	Most	Least	Most	Least	Most	Least	Most	Least	Most	Least
*TIP41*	*EF1α*	*SAND*	*EF1α*	*TIP41*	*SAND*	*GAPDH*	*EF1α*	*GAPDH*	*EF1α*	*TIP41*	*EF1α*	*F-box*	*SAND*	*CACS*	*SAND*	*TIP41*	*CACS*
*GAPDH*		*TUB*		*TUB*		*TIP41*		*TUB*		*PEPKR1*		*TIP41*		*TUB*		*TUB*	
*ACT*												*TUB*				*ACT*	

### Reference gene validation for a target gene, *FaWRKY1*, expression

**Fig 5:**
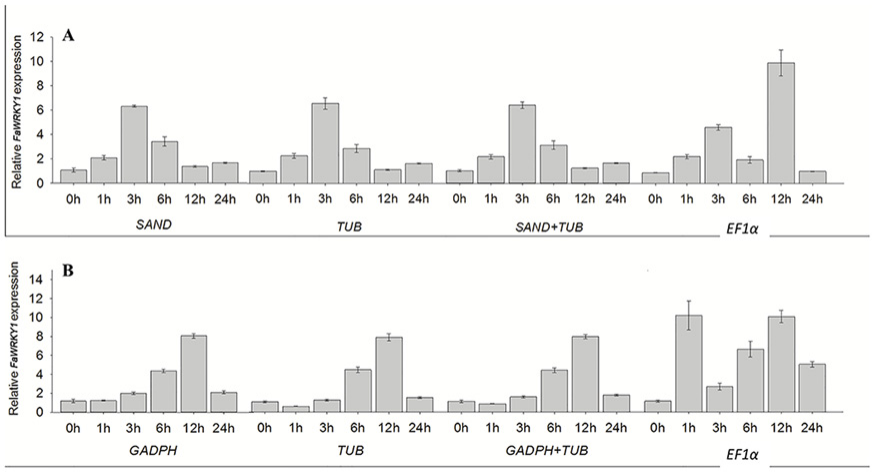
Relative expression of *FaWRKY1* using selected reference genes including the most (*SAND* and *TUB*) and the least (*EF1α*) stable reference genes for normalization, following the stress treatment after 0, 1, 3, 6, 12 and 24 h. (A) *FaWRKY1* expression of roots under salt-stress; (B) *FaWRKY1* expression of leaves under drought-stress (PEG treatment). The error bars represent standard error.

To confirm utility of candidate reference genes, the expression pattern of a target gene, *FaWRKY1* in response to salinity and drought stress in SR and PL samples was determined (Fig. 5). The two most-stable references genes (*SAND* and *TUB*) for SR samples and *GAPDH* and *TUB* for PL samples, and the least-stable reference gene (*EF1α*) selected from the analyses previously described were used in the validation test. Using *SAND* or *TUB* alone or *SAND* combined with *TUB* as the reference genes, the *FaWRKY1* expression in roots of tall fescue increased during salt stress to reach a peak at 3 h and then declined with longer duration of stress. When normalized by *GAPDH* or *TUB* alone or *GAPDH* combined with *TUB*, the *FaWRKY1* expression level increased to the highest level by 12 h of drought stress in leaves and then decreased with longer duration of stress (Fig. 5a, b). The expression level of *FaWRKY1* assayed with *EF1α* as a reference gene exhibited fluctuations and failed to achieve a consistent pattern in response to either salinity or drought (Fig. 5a, b).

## Discussion

Several programs, such as geNorm, NormFinder and BestKeeper [18,19] have been successfully employed to determine the stability of reference gene expression and identify stable reference genes for various plant species, such *Populus euphratica* [8], *Lolium perenne* [13], *Phaseolus vulgaris* [27] and *Cynodon dactylon* [28]. To our knowledge, this is the first comprehensive study which identified stable reference genes for normalization of qRT-PCR analysis of target gene expression in leaves and roots of tall fescue exposed to four abiotic stresses (salinity, drought, heat, and cold) utilizing four different methods (GeNorm, NormFinder, BestKeeper and RefFinder) with RefFinder integrating results from GeNorm, NormFinder, BestKeeper. The comprehensive analysis with four programs identified stable reference genes for qRT-PCR of target gene expression in leaves and roots of tall fescue under different abiotic stress and indicated that nine candidate reference genes exhibited differential stability in leaves and roots.

Several genes, including *ACT*, *TUB*, *GAPDH* and *EF1α*, have been commonly used as the reference genes for gene expression assays in different plant species, but exhibited variable expression among different species or tissues or environmental conditions [9,10,13]. *EF1α* exhibited stable expression in soybean (*Glycine max*) under drought and salinity stress [29], in *Populus euphratica* under cold treatment [8], and in *Caragana korshinskii* under heat stress [6]. However, in this study, *EF1α* was the least-stable in both leaves and roots of tall fescue exposed to all four abiotic stresses (Table 5). *GAPDH* exhibited stable expression in heat-treated buffalograss (*Buchloe dactyloides*) [30], but unstable expression in rice under various environmental conditions [31]. In our study, *GAPDH* exhibited stable expression in both leaves and roots of tall fescue under drought stress. *ACT* and *TUB* were recommended for use in salinity and drought stress in *Vigna mungo* [32], whereas a unstable expression of *ACT* and *TUB* under four abiotic stresses was found in *Caragana intermedia* [9]. In this study, TUB could be used as a reference gene for qRT-PCR normalization of salnity- and heat-treated roots and leaves. ACT was one of the most commonly-used reference genes in previous studies [27,29,30], but its stability varied among samples examined and with the methods of analysis in this study (Table 5).

Some genes, such as *TIP41*, *SAND*, *CACS*, *F-box*, *PEPKR1*, from *Arabidopsis* microarray data were suggested as references genes [5] and showed more-stable expression than some commonly-used reference genes, such as *ACT*, *TUB*, *GAPDH* and *EF1α* in stress-treated *Caragana intermedia* and buckwheat of different developmental stages [8,9]. *TIP41* and *PEPKR1* were used as two reference genes for normalizing gene expression data across various tissues (leaves, stems, cotyledons, hypocotyls, and roots) in peanut (*Arachis hypogaea*) [33], or PEG- and heat-treated leaves in *Caragana intermedia* [9]. *SAND* and *CACS* were used as reference genes in a variety of developmental and environmental conditions in buckwheat (*Fagopyrum esculentum*) [11]. A recent study reported that *F-box* could be used for normalization of cold-stressed or salicylic acid-treated *Brassica napus* and for different tissues, organs, and developmental stages of *Litsea cubeba* [34,35]. Our study also demonstrated that *TIP41*, *SAND*, *CACS*, *F-box* and *PEPKR1* could be suitable for gene normalization of both leaves and roots under four different abiotic stress in tall fescue (Table 5).

Significant variations of target gene levels were found when unstable reference genes were used as the internal control, leading to misinterpretation of experimental results [9,34]. In this study, SAND and TUB or GAPDH and TUB were found to be suitable for the quantification of FaWRKY1 expression patterns in salinity-stressed roots or drought-stressed leaves of tall fescue, because the target gene, *FaWRKY1* exhibited clear expression patterns in response to salinity or drought stress using SAND and TUB or GAPDH and TUB as the internal reference. The most unstable *EF1α* selected using GeNorm, NormFinder, BestKeeper, and RefFinder was confirmed to produce unreliable qRT-PCR results, as shown by fluctuating expression patterns of *FaWRKY1* expression in roots and leaves of tall fescue in response to salinity or drought stress. Variable expression patterns of *FaWRKY1* in response to salinity and drought stress resulting from the use of different references genes in this study indicated that the appropriate selection of reference genes serves important roles for normalization of target gene-expression data with qRT-PCR.

In summary, either the combination of SAND and TUB or TIP41 and TUB could be used as stable reference genes for qRT-PCR quantification of target genes in salinity-treated roots and leaves. The combinations of *GAPDH* with *TIP41* or *TUB* were suitable for gene quantification of roots and leaves under drought stress. *TIP41* and *PEPKR1* maintained stable expression in cold-treated roots, and the combination of *F-box*, *TIP41* and *TUB* could be applied for cold-treated leaves. *CACS* and *TUB* were the two most-stable reference genes in heat-stressed roots, *TIP41* combined with TUB and *ACT* were recommended for heat-stressed leaves. Different combinations of reference genes were recommended to be effective internal controls for quantify target gene expression with qRT-PCR in tall fescue under different abiotic stresses. The stable reference genes identified in this report will enhance accuracy of normalization and quantification of target gene expression with qRT-PCR analysis for tall fescue under different abiotic stresses and facilitate the identification of stress-responsive genes and molecular mechanisms conferring stress tolerance in this species.
